# A link prediction-based recommendation system using transactional data

**DOI:** 10.1038/s41598-023-34055-5

**Published:** 2023-04-27

**Authors:** Emir Alaattin Yilmaz, Selim Balcisoy, Burcin Bozkaya

**Affiliations:** 1 Faculty of Engineering and Natural Sciences, Sabanci University, Istanbul, Turkey; 2 Sabanci Business School, Sabanci University, Istanbul, Turkey

**Keywords:** Mathematics and computing, Computer science

## Abstract

Recommending relevant items to users has become an important task in many systems due to the increased amount of data produced. For this purpose, transaction datasets such as credit card transactions and e-commerce purchase histories can be used in recommendation systems to understand underlying user interests by exploiting user-item interactions, which can be a powerful signal to perform this task. This study proposes a link prediction-based recommendation system combining graph representation learning algorithms and gradient boosting classifiers for transaction datasets. The proposed system generates a network where nodes correspond to users and items, and links represent their interactions. A use case scenario is examined on a credit card transaction dataset as a merchant prediction task that predicts the merchants where users can make purchases in the next month. Performances of common network embedding extraction techniques and classifier models are evaluated via various experiments conducted and based on these evaluations, a novel system is proposed, and a matrix factorization-based alternative recommendation method is compared with the proposed model. The proposed method has shown superior performance to the alternative method in terms of receiver operating characteristic curves, area under the curve, and mean average precision metrics. The use of transactional data for a recommendation system is found to be a powerful approach to making relevant recommendations.

## Introduction

Graphs are data structures that can powerfully abstract complex systems. Many real-world systems can be represented in a graph structure where individuals and relationships between them correspond to nodes and edges. These systems include social networks^1,2^, biological networks^3,4^, physical networks^5^, citation networks^2^, knowledge representations^6^ and much more^7^. Due to its applicability in a wide range of domains, graph systems advancements can lead to great improvements in many diverse fields.

The systems that exploit network structures can perform common tasks such as node classification^8^, community detection^9^, and link prediction^10^. Node classification is to decide the class of a node in a graph; community detection is to find the clusters that contain similar nodes in a network, and link prediction is to discover hidden links based on observed links.

Link prediction has many diverse applications in real-world scenarios such as predicting the future friends in a social network^11^, detecting future collaborations in an academic network^2^, finding protein–protein interactions (PPI) in a biological network^12^, and recommending items in recommendation systems^13^. Due to its high practicality, research in this field can lead to many contributions to a wide range of systems. Among these applications, recommendation systems are the primary consideration of this study.

Recommendation systems offer items to users that they might be interested in. These items can be relevant links on a search engine, products on an e-commerce website, movies, music, and much more. Common recommendation systems use collaborative filtering-based approaches that utilize a user-item matrix to make recommendations using users’ past activity. However, these methods may have sparsity and scalability issues^14^. A link prediction approach for a recommendation system can operate in a small network neighborhood which can be proposed as a scalable solution for the scalability issues in recommendation systems.

Due to advancements in information technologies, the availability of customer transaction data has increased^15^. Hence, this provides an opportunity to have more insights into consumer purchase behavior, which plays a huge role in determining marketing strategies. Moreover, it is also useful for detecting the target market, which has many implications for increased sales. Understanding the audience can help merchants to create personalized recommendations, advertising campaigns, promotions and allocate resources efficiently^16,17^. In classical methods, audience demographics extracted from census data, psychographics, and customer surveys are used for segmentation of customers to the target audience^18^. In addition to these statistics, product categories or market-basket analysis methods are also used to understand customer purchase preferences. However, their ability of customer purchase prediction is limited^19^. Transaction datasets consisting of records with item-user pairs, timestamps, and optional numerical quantities can be exploited to understand user interests and make recommendations. Such datasets can be obtained from many fields in different forms, such as credit card transactions, e-commerce purchase history, user click history on web pages, songs listened to or movies watched on a streaming service, all of which can be interpreted as transactional data. A system based on the use of transactional datasets can be diversely applied in many fields.

To provide further context, the proposed method aims to address the challenge of link prediction in recommendation systems. This is a critical problem as accurately predicting the links between users and items can greatly improve the effectiveness of recommendations, leading to increased user satisfaction and revenue for businesses. However, traditional approaches to link prediction often rely on explicit feature engineering, which can be time-consuming and may not capture all relevant information. We propose a link prediction-based recommendation system using a transactional dataset with these motivations. Our goal is to provide a framework using a transactional dataset that helps predict and recommend relevant items which the user can potentially interact but was not previously linked with. Overall, the proposed method aims to provide a more efficient and effective solution to the link prediction problem in recommendation systems, by leveraging the power of node embeddings and machine learning techniques.

The proposed method in this study adopts a binary classification approach for a link prediction problem where the actual links and non-existent links are classified as “true” and “false”, respectively. This task includes a preprocessing step, an embedding model, and a classifier model. During the preprocessing step, transaction data is processed to create a graph network where nodes are users and items, and links are the associations between them. The embedding model extracts the network information and puts it in a low dimensional form (i.e., *node embeddings*) which can be used in machine learning tasks. A binary operator converts extracted node embeddings into a link embedding form. Then, embeddings of existent and non-existent links are used to train a classifier model, and then this model is used to classify a link as “true” or “false” by thresholding. As a use case scenario, a credit card transaction dataset is used to predict and recommend merchants which customers might visit to make purchases in the next month based on their past transactions.

The main contributions of this study are as follows:

- A link prediction-based recommendation system exploiting transactional data is proposed, and using transactional data for a recommendation task is found to be a powerful signal.
- Performances of embedding extraction methods node2vec^20^, metapath2vec^21^ and binary operators averaging, hadamard, l2 and classifier models XGBoost^22^, LightGBM^23^, Artificial Neural Networks are compared to create a link prediction system and used on a credit card transaction dataset as a use case scenario. Based on the evaluations of the area under the curve (AUC) and mean average precision at K (MAP@K) metrics, metapath2vec^21^, averaging, LightGBM^23^ is found to be the best combination and presented as the proposed model.
- Performance of a collaborative filtering-based alternative recommendation method Alternating Least Squares^24^ is compared with the proposed model. Our proposed method is shown to have superior performance to the alternative method in terms of receiver operating characteristic (ROC) curves, the area under the curve (AUC), and MAP@K scores.

## Related works

### Link prediction algorithms

There are several approaches for the link prediction task. The most commonly used are similarity-based algorithms which assume that two similar nodes will interact with a high probability^25^. These methods assign a similarity score for a pair of nodes and create an ordered list of links based on these scores. Although this is a reasonable approach, node features may not always be present. Learning-based models adopt a probabilistic approach that generates a joint probability distribution that abstracts the observed graph by optimizing an objective function with certain model parameters. Based on this distribution, link existence and non-existence probabilities can be calculated using conditional probability^26–28^. A learning based model can be obtained by graph representations that use these conditional probability approaches.

### Graph representation learning algorithms

Graph representation learning algorithms are an essential part of link prediction methods. These algorithms use graph representations in a low-dimensional space by capturing network information. These low dimensional feature representations can be used in machine learning tasks such as anomaly detection, attribute prediction, clustering, and link prediction^29^, which is directly related to our study and is worth examining. The classical methods of network feature extraction rely on network properties such as node degrees^30,31^, but these methods also conduct a hand-crafted feature extraction process, which is not practical in many cases. Unsupervised representation learning algorithms do not involve hand-crafted feature extraction and make the whole process automated. Recent advancements in natural language processing (NLP) techniques have opened a new path for representation learning. One of the noteworthy NLP methods is Word2vec^32^ which creates vector representations of words from a large corpus where similar words are put in the same or similar context. The skip-gram architecture in this technique uses the current word to predict the nearby words in a window of context words which lead some graph representation algorithms to treat a graph as a large corpus and create random walks around each node representing sentences to learn latent representations. Similar to graph representation methods such as DeepWalk^33^ and LINE^34^, node2vec^20^ combines both bread-first and depth-first search strategies and performs biased random walks around nodes. Despite having convincing performances of graph feature extraction methods mentioned in both homogeneous and heterogeneous graphs, these methods are mainly developed to be used in homogeneous graphs. Metapath2vec^21^ is developed for heterogeneous graphs to have better representation by considering heterogeneity, which takes node types into account while creating random walks. Random walks are generated based on meta-path schemes to create heterogeneous neighborhoods for different types of nodes. The goal of this method is to maximize the likelihood of heterogeneous network neighborhood. Therefore, using unsupervised representations in a link prediction context may be a powerful approach since it is applicable to a wide range of datasets represented by a graph structure associated with the link prediction problem.

### Link prediction as a recommendation system

Many studies use graph structures and link prediction methods as recommendation systems. Some common similarity measures^14^, resource allocation-based approaches^35^, random walk-based recommendation frameworks simulating a friend hunting behaviour^36^ are seen to be used in the recommendation system context. A link prediction based recommendation approach^37^ creates a graph where nodes are users and items, and links are the associations between them with weights as complex numbers to assess user similarity and user-item interest information. Another study on friend recommendation in social networks^38^ extracts social patterns from different networks and transfers this information as attribute correlation and social correlation into a Markov Random Field while recommending links. Most of the link prediction-based recommendation system methods^39,40^ are various adaptations of existing link prediction algorithms discussed above.

While many studies use graph structures and link prediction methods as recommendation systems, our proposed method focuses on leveraging node embeddings and machine learning techniques to predict relevant links and recommend items that users may interact. This approach is different from traditional methods^10,41^ that rely on explicit feature engineering and may not capture all relevant information. Additionally, our method involves converting transactional data to a graph structure and utilizing this signal, which is a notable difference from previous works^10^ in the field.

## Problem formulation

Consider a graph $G=(N,L)$, where *N* is the set of nodes and *L* is the set of links. Let *L* represent the “true” link set in the network and consider the case when only some parts of the links are given. These given links are the “observed” links and denoted as $L_O$ where $L_O \subseteq L$. The task is to discover the hidden (unobserved) link set $L_H$ where $L_H \cup L_O = L, L_H \cap L_O = \emptyset$. For the temporal formulation of the link prediction problem, let $L_{t}$ be the set of true links in a network seen at time *t*. The goal is to predict $L_{t+1}$ which is the set of true links at time $t+1$.

## Methods

In this study, the research task is formulated as a link prediction problem. In this context, a *heterogeneous* graph that contains different types of nodes and links is generated from the transactions where nodes correspond to users and items, and links represent interaction occurrences between these users and items. Given such a network generated from past transactions, the goal is to discover new possible user-item link formations in a future network version. Since the problem investigates link occurrences between pairs of nodes, it can also be formulated as a binary classification task to detect if there is a link or not. Links between nodes are considered “true” links if there is a real connection among examined nodes and “false” links if there is no actual connection between these nodes in the network. A classifier is trained, which then tries to predict if a given test link is true or not. In this formulation, probability estimates of possible links can be associated with their probability of existence.

**Figure 1:**
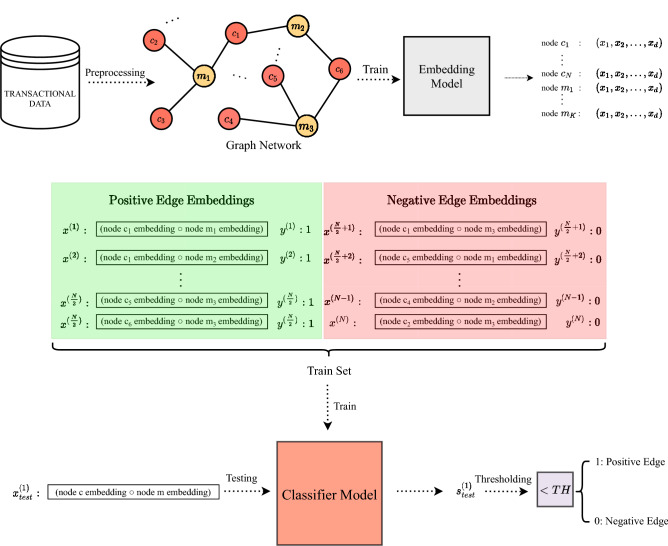
Illustration of proposed method.

At the preprocessing step, transaction data is cleaned and processed to create a graph architecture where nodes correspond to users and items, and links represent their interactions. An embedding model captures network information and extracts low dimensional latent space representations of nodes (embeddings) that can be used as inputs to machine learning systems. A binary operator is applied to node embeddings to form link embeddings. Then, embeddings of existent and non-existent links train a classifier model which will be able to classify a link as “true” or “false” after applying suitable thresholds. Proposed method is illustrated in Fig. 1.

### Node embeddings

In order to benefit from the expressive power of graph architectures in machine learning tasks, there is a requirement to transform their complex topologies into d-dimensional vector representations before giving them as input to machine learning models. These low dimensional embeddings are generated and examined in terms of their performances using common node embedding extraction methods in the literature, namely node2vec^20^ and metapath2vec^21^. Our embedding model is trained by nodes and corresponding positive links, which capture the network information.

The motivation behind using node embeddings is that to capture hidden connections between nodes by representing them as low-dimensional vectors in a continuous space, allowing for more efficient processing and analysis of graph data. In addition, node embeddings can be an effective approach in addressing the “cold start” problem in recommendation systems, where new nodes without any neighborhood information can be easily incorporated into the embedding space by estimating their embeddings based on their external features^42^. For example, if a new node is added to a recommendation system, the system can estimate the embedding of the node based on its external features, such as demographic information for a user, interests, and preferences or metadata, such as title, description, category for items. Estimating the embedding of a new node allows for effective incorporation of the node into the embedding space, while preserving neighborhood information that can be leveraged to capture the relationships of the node with existing nodes in the graph.

#### node2vec

Node2vec^20^ optimizes a graph-based objective function by the stochastic gradient descent algorithm. The embedding extraction method relies on maximizing the likelihood of preserving the network neighborhood. To achieve this goal, 2nd order biased random walks are used to create network neighborhoods of the nodes.

To perform a biased random walk, unnormalized transition probabilities are calculated as the multiplication of the static edge weights by a bias factor $\alpha$. So, when the walker at node *v* came from node *t*, the next node *x* of the random walk is evaluated based on this probability ($\pi _{vx}$):

$$\begin{aligned} \pi _{vx} = \alpha _{pq}(t,x) \cdot w_{vx} \end{aligned}$$

where $w_{vx}$ is the static edge weight between node *v* and node *x* and bias factor $\alpha _{pq}$ with second order random parameters *p* and *q* guiding the walk is defined as follows:

$$\begin{aligned} \alpha _{pq}(t,x) = \left\{ \begin{aligned}&\frac{1}{p},{} & {} \text {if}\ d_{tx} = 0 \\&1,{} & {} \text {if}\ d_{tx} = 1 \\&\frac{1}{q},{} & {} \text {if}\ d_{tx} = 2 \\ \end{aligned} \right. \end{aligned}$$

where $d_{tx}$ is the distance between node *t* and node *x*.

#### metapath2vec

metapath2vec^21^ is designed to apply on heterogeneous graphs which contain different types of nodes. Furthermore, since the user-item network is a heterogeneous graph, metapath2vec^21^ is also examined. This method uses a random walk approach as node2vec^20^ and LINE^34^; in comparison with them, it then performs meta-path-based random walks to include heterogeneity.

Formally, given a heterogeneous network $G=(N,L,T)$, the mapping functions associated with node *n* and link *l* are defined as follows: $\phi (n): N \rightarrow T_N$ and $\phi (l): l \rightarrow T_L$. $T_N$ is the set of node types and $T_L$ is the set of link types, and $|T_N|+|T_L| > 2$. Our method learns d-dimensional node embeddings $\textbf{X} \in {\mathbb {R}}^{|N|\times d}$. Here $\textbf{X}$ is a matrix and $X_n$ corresponds to the d-dimensional vector embedding of node *n*. This algorithm maximizes the network neighborhood probability of the corresponding type of nodes that preserves the context information for each node type.

For a heterogeneous graph $G=(N,L,T)$ and meta path $P: N_1 \xrightarrow {R_1} N_2 \xrightarrow {R_{2}} \cdots N_t \xrightarrow {R_t} N_{t+1} \cdots \xrightarrow {R_{k-1}} N_k$, where $R = R_1 \circ R_2 \circ \cdots \circ R_{k-1}$ defines a composite relation between node types $N_1$ and $N_k$, $\circ$ is composition operator, the transition probability *p* for a random walk at step *i* based on a meta-path scheme *P* is determined as the following:

$$\begin{aligned} p(n^{i+1}|n_t^{i},P) = \left\{ \begin{aligned}&\frac{1}{|S_{t+1}(n_t^i)|}{} & {} \ (n^{i+1},n_t^i) \in L, \phi (n^{i+1}) = t+1 \\&0{} & {} \ (n^{i+1},n_t^i) \in L, \phi (n^{i+1},n_t^i) \ne t+1 \\&0{} & {} \ (n^{i+1},n_t^i) \notin L \\&1{} & {} \text { if } t = 1 \\ \end{aligned} \right. \end{aligned}$$

where $n_t^i \in N_t$ and $S_{t+1}(n_t^i)$ is $N_{t+1}$ type of network neighborhood of node $n_t^i$.

### Link embeddings and binary operators

To perform a link prediction task, a “link” formulation is also needed in the machine learning framework. An edge/link is the connection between a pair of nodes. However, from an input perspective for the prediction task, it is the combination of the embeddings of the nodes that are interacting with each other by a binary operator. A binary operator $\odot$ generates a link representation *g*(*u*, *v*) such that $g: V \times V \rightarrow {\mathbb {R}}^{d}$ where *d* is the vector size based on node embedding vectors *f*(*u*) and *f*(*v*) of node *u* and node *v*. This operator is intended to have a generalization power which also enables the representation of non-existent “false” or “negative” links.

In order to preserve vector dimension *d* in these “link embeddings”, three most common binary/element-wise operators are considered, namely *average*, *hadamard* and *l2*^20^ shown in Table 1.

**Table 1:** Binary operators and definitions adopted from^20^.

Binary operator	Symbol	Definition
Average	$\boxplus$	$[f(u) \boxplus f(v)]_{i}=\frac{f_{i}(u)+f_{i}(v)}{2}$
Hadamard	$\circ$	$[f(u) \circ f(v)]_{i}=f_{i}(u) * f_{i}(v)$
L2	$\Vert \cdot \Vert _{2}$	$\Vert f(u) \cdot f(v)\Vert _{{\overline{2}}i}=\left| f_{i}(u)-f_{i}(v)\right| ^{2}$

### Data partitioning and negative sampling

Our transactional dataset is partitioned according to a timestamp. Transactions before a certain timestamp are used to form a heterogeneous graph on which an embedding model is trained, and link embeddings are generated. These embeddings form the positive links of the training set used by the classifier model that seeks binary classification. In addition to that, the negative links, links that are not existent in the network, are also present in this training set for the classifier to be trained robustly. A sampling of the negative links is achieved by randomly selecting a pair of nodes that are not linked in the network and generating its link embedding by applying a binary operator. To have a balance in both classes, a number of positive and negative links are selected as equal. Since most real networks are sparse, there are many non-existing links. Thus, generating negative samples is not a complex process.

In addition, a hyperparameter tuning step is included to select the best embedding and classifier models to perform the task. This step is achieved by partitioning data further into a validation set, again splitting by another timestamp. The same processes also obtain the test set. To illustrate, with the transactions in an *M*-month period, the first $M-2$ months can be used for training, $M-1$st month for validation, and the last month in the dataset as the test set for evaluating the models. After the model selection steps, the train and validation sets are merged to train the best classifier to apply on the test set. From the *M*-month transaction data, the validation and test sets include some users and their $M-2$nd and $M-1$th-month transactions as positive links, respectively. On the other hand, since the task is to discover the newly formed transactions in this month, one should consider all the item combinations for a specific user. Simply taking the only positive links of a user for this month is not the right approach because this information is unknown and needs to be discovered.

For a fair evaluation, given one user, the interactions between this user and all items must be checked where actual transactions are labeled as positive and non-existent interactions as negative links in validation and test sets since they are not included in the training set. The classifier model should then be able to differentiate which links are positive or negative.

### Classifier model

Various machine learning models are examined and compared in terms of performance due to their different characteristics and predictive potential. Gradient boosting algorithms and artificial neural networks have high predictive powers and hence are considered for the binary classification task. Positive and negative link embeddings train the classifier model.

#### XGBoost

XGBoost^22^ (eXtreme Gradient Boosting) is an algorithm based on the gradient boosting concept. It introduces a regularization term that reduces overfitting. Due to parallelized and distributed structure of XGBoost also reduces the training times.

#### LightGBM

Microsoft developed a gradient boosting framework LightGBM^23^ to get more efficiency while working on big data by feature selection and decreasing the data size. The Exclusive Feature Bundling (EFB) technique regroups the mutually exclusive features into bundles and takes them as a single feature, which reduces the number of features. Gradient-based One-Side Sampling (GOSS) keeps the data points that cause larger gradient changes during data sampling to gain more information.

#### Artificial neural networks

As a classifier model, a two-layered feed-forward neural network with sigmoid and ReLU activation functions is also considered for the classification task.

### Alternative method

Alternating least squares (ALS)^24^ is chosen as the alternative method, as it has been used in many studies^43–45^ and is considered a strong benchmark for latent factor model type of collaborative filtering .The number of times a user interacts with an item (forms a link) is used as the implicit information. This implicit information is used since the transactional datasets are not generally explicit, which does not provide rating information that is used in collaborative filtering methods. Alternating Least Squares (ALS)^24^ method is a case of the matrix factorization technique.

An interaction matrix *R* is defined for implicit datasets, representing user and item interactions. The main idea behind the ALS method is to factorize this interaction matrix *R* into smaller matrices *U* as user features and *V* as item features, with $r_{ui}$ being the interaction value of user *u* with item *i*. The preference $p_{ui}$ of user *u* on item *i* is calculated based on the interaction value $r_{ui}$ as follows:

$$\begin{aligned} p_{u i}=\left\{ \begin{array}{ll} 1 &{} r_{u i}>0 \\ 0 &{} r_{u i}=0 \end{array}\right. \end{aligned}$$

In this method, to find the optimal user interaction and item interaction matrices, an alternative optimization method is adopted instead of the stochastic gradient descent algorithm to avoid the cost function’s costly computations that combine the mean squared errors of matrices and the regularization term. By fixing user and item interaction matrices and taking derivatives alternatively, the cost function becomes quadratic, which can be optimized by the Alternating Least Squares method. This process continues iteratively, where the value of the cost function is improved at each step.

## Use case

As a use case scenario merchant prediction task is performed. Merchant prediction refers to discovering the potential merchants where a customer can visit and make a purchase. This discovery process provides detailed insights into consumer purchase behavior, which plays a massive role in determining marketing strategies. The transaction dataset has these customer-merchant pairs of information that can predict future purchases. In this context, the link prediction-based methodology developed in this study is used to discover merchants where a customer can make a purchase but has not visited before. These predictions can then be used as recommendations. A use case scenario of our proposed link prediction model will be presented with a real-world transaction dataset.

### Dataset description and data partitioning

**Figure 2:**
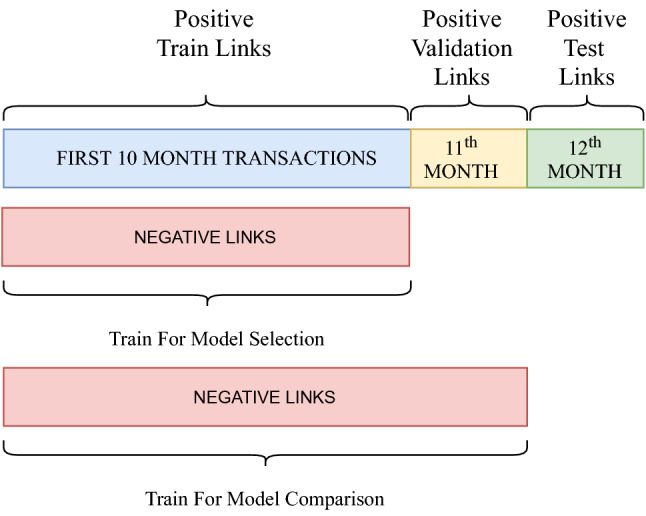
Data partition visualization.

We used an anonymized credit card transaction dataset, provided by a private bank in an OECD country, which is a real-world dataset that has been employed in various studies^46,47^ in the field. The columns represent customer id, transaction time, transaction amount, merchant id, merchant category, and the latitude and longitude coordinates of the merchant location. The dataset includes 627.184 transactions made by 51.451 customers at 482 merchants from July 2014 to June 2015. The vast majority of transactions are made in this OECD country’s largest and most populous metropolitan city. The transaction dataset is preprocessed to create the merchant-customer network where nodes are customers and merchants, and links represent the purchase occurrence between them. As defined in our proposed model architecture, the dataset is split as train, validation, and test sets. The train set contains transactions from the first ten months, the validation set from the 11th month, and the test set from the 12th month with positive links. In addition, an equal number of negative transactions are randomly generated and added to the train set. On the other hand, for validation and test sets, since the task is to discover newly formed transactions in those months, all merchant-customer combinations are considered for a specific customer as negative links. Viusal representation and details of data partition can be seen in Fig. 2 and Table 2, respectively.

### Embedding extraction

To represent the merchant-customer network in an embedding space, node2vec^20^ and metapath2vec^21^ algorithms are utilized with specified parameters for the embedding space dimensionality, random walk length, and number of walks per node. After setting the parameters, random walks are generated from each node, following the transition probabilities defined by the respective algorithms. Specifically, metapath2vec^21^ model utilized with the meta-path scheme of customer $\rightarrow$ merchant $\rightarrow$ customer. Table 4 shows the details of the parameter settings used for both embedding models.

**Table 2:** Data partition details.

Data	# Pos. links	# Neg. links	# Customers	# Merchants
Train	119,931	119,931	41,431	397
Validation	8410	5,777,942	14,723	397
Train + validation	132,410	132,410	43,245	426
Test	7165	5,743,713	13,640	426

## Evaluation

In order to decide which methods mentioned in the architecture design are superior, we performed an evaluation process. This process consists of the embedding model, the classifier model, and the binary operator selection stages. In addition, hyperparameter tuning is conducted for each model. To evaluate and compare the performances of these models, receiver operating characteristic (ROC) curve, mean average precision at K (MAP@K), and area under curve (AUC) metrics are used in this study.

Mean average precision at K (MAP@K) metric treats the recommendation system as a ranking task since recommendation systems offer a ranked list of *K* items based on their recommendation score. This score can be similarity, probability, or any other measurement.

$$\begin{aligned} AP@K = \frac{\sum _{k=1} ^K P(k) \times rel(k)}{R} \end{aligned}$$

where *AP*@*K* is average precision at *K*, *R* is the number of relevant items, *P*(*k*) is precision for the first *k* ranked items, *rel*(*k*) is an indicator function equals to 1 if the item at rank *k* is relevant, 0 otherwise.

$$\begin{aligned} MAP@K = \frac{\sum _{q=1} ^Q AP@K_q}{Q} \end{aligned}$$

where *Q* is the number of queries, $AP@K_q$ is the average precision at *K* value for this query *q*. In the merchant prediction context, *Q* is the number of customers being considered in the validation or test set, and *R* is the number of new purchases that need to be discovered for a customer. *AP*@*K* value is calculated for each user and taking the average of these values gives the *MAP*@*K* value.

Mann–Whitney U-Test^48^ is used to examine the significance of the results. Since prediction scores are divided into positive and negative link groups, samples taken from these groups will be used in the U-Test. This test will examine whether positive link scores are significantly higher than negative link scores or not.

## Experiments

To evaluate the effectiveness of our proposed model, numerous experiments are conducted. In the first set of experiments, the training set (the first 10 months of transactions) and validation set (11th month transactions) are used to decide which embedding model, binary operator, and the combination is the best. There are a total of 72 different combinations in the experiments. After the decision, the best combination is used on the test data to compare our approach with the alternative method, the Alternating Least Squares (ALS)^24^. The training data used in comparison consists of merging the train and validation sets. For the embedding model hyperparameter tuning part, number of walks and walk length are the predominant hyperparameters that affect the embedding model performances. Therefore, the effects of different values of these hyperparameters are examined. For other configurations, the meta-path used in the metapath2vec^21^ model is customer $\rightarrow$ merchant $\rightarrow$ customer, and the embedding dimension is 100. There are eight different embedding models present in the experiments. In order to decide on the classifier model, the models mentioned above with default parameter values in their libraries are considered in the selection process. All classifier models, classifier models and binary operators used in this work is summarized in Table 3. Naming of the embedding models with their model hyperparameters and classifier model parameter configurations are detailed in Tables 4 and 5, respectively.

**Table 3:** Summary of models and operators to be selected.

Embedding models	Classifer models	Binary operators
node2vec^20^	XGBoost^22^	Average
metapath2vec^21^	LightGBM^23^	Hadamard
	ANN	L2

**Table 4:** Embedding model hyperparameters.

Model name	Embedding type	Number of walks	Walk length
N1	node2vec^20^	1	100
N2	node2vec^20^	1	200
N3	node2vec^20^	10	100
N4	node2vec^20^	10	200
MP1	metapath2vec^21^	1	100
MP2	metapath2vec^21^	1	200
MP3	metapath2vec^21^	10	100
MP4	metapath2vec^21^	10	200

**Table 5:** Classifier model parameter configurations.

Model name	Parameters
LGBM^23^	objective: binary, is_unbalance: true, feature_fraction: 0.5, bagging_fraction: 0.5, bagging_freq: 20, num_boost_round = 500
XGB^22^	max_depth: 11, eta:0.3, objective: binary logistic, max_bin:16, num_rounds: 500
ANN	hidden_units: 32, input_dimension:100, optimizer: adam, loss: binary cross entropy, activations: reLu, sigmoid

## Results

### Validation results

**Table 6:** Top-10 validation experiment results.

Rank	Classifier	Embedding model	Operator	AUC	MAP@5
1	LGBM	MP2	Avg	0.842366	0.065144
2	LGBM	MP1	Avg	0.867941	0.064438
3	LGBM	N1	Avg	0.86557	0.064115
4	LGBM	N2	Avg	0.83629	0.063995
5	LGBM	N1	Hadamard	0.860167	0.061063
6	XGB	N1	Avg	0.85034	0.060578
7	LGBM	MP2	Hadamard	0.85298	0.060059
8	XGB	MP2	Avg	0.833313	0.059653
9	LGBM	MP1	Hadamard	0.859542	0.05955
10	XGB	MP1	Hadamard	0.845396	0.059514

#### Classifier model

Table 6 shows the top-10 experiment results among 72 experiments sorted according to MAP@5 values on the validation set. The best MAP@5 score is 0.065144 with LGBM^23^ + metapath2vec^21^ + average operator. The LightGBM^23^ classifier model is dominant in the top 10 ranked experiments. XGBoost^22^ classifier is the second best in the rankings. ANN comes into the picture after the 18th experiment, and it shows the worst performance among the three classifiers.

According to these results, it can be inferred that LGBM^23^ creates more complex decision trees than XGBoost^22^, giving better results. Also, ANN prediction scores are close to each other, possibly concentrated near 1 or 0, which does not create a powerful, distinguishing feature for a ranking task.

#### Embedding model

For the embedding model selection, models with metapath2vec^21^ embeddings show better performance in the first two experiments. Although models with node2vec^20^ embeddings have convincing results in the next set of experiments, due to the heterogeneous nature of the network built in this study, it is not surprising that models with metapath2vec^21^ embeddings produce better results than others. Also, walk length on heterogeneous networks seems to be an essential hyperparameter by examining the orderings of MP1 and MP2. In most experiments, MP2 has a superior performance (has higher orders) than MP1 while fixing other features. This situation is the opposite in node2vec^20^ embeddings (for homogeneous networks); comparing N1 and N2 embeddings, N1 has superior performance to N2. Furthermore, an increasing number of walks can cause overfitting and make the performance worse based on these results.

#### Binary operator

Among the binary operators, the *average* operator is the best as it dominates the first four experiments with the highest MAP@5 values. The *Hadamard* operator is the second-best operator after *average* based on its presence in the top 10 sets of experiments. *L2* operator seems to have the worst performance among the three. Since the node embeddings represent coordinate values in the latent space, it is reasonable to take element-wise averages in a geometrical perspective to preserve information of both nodes to form a link.

There are only minor changes in AUC scores throughout the experiments. Moreover, there is an imbalance between positive and negative links in the test set, where positive links are only a small portion of the entire test set. Because of this, models can predict the negative values more efficiently, which increases the AUC score. Therefore, it can be interpreted that AUC scores are not so significant in differentiating between the validation models. However, we find the variation higher with MAP@5 values. Hence we ranked the experiments in the decreasing order of their MAP@5 values.

### Test and comparative results

We compare the proposed and alternative models according to MAP@5 and AUC scores with the test set. Also, we calculate the U-Test statistics to examine the significance of the results which can be seen in Table 7.

**Table 7:** Comparisons of the proposed and alternative methods.

Model	AUC	MAP@5	Mean U-test statistics
Proposed	0.8557	0.064718	331.421
Alternative	0.6994	0.047719	278.145

#### Receiver operating characteristic curves

**Figure 3:**
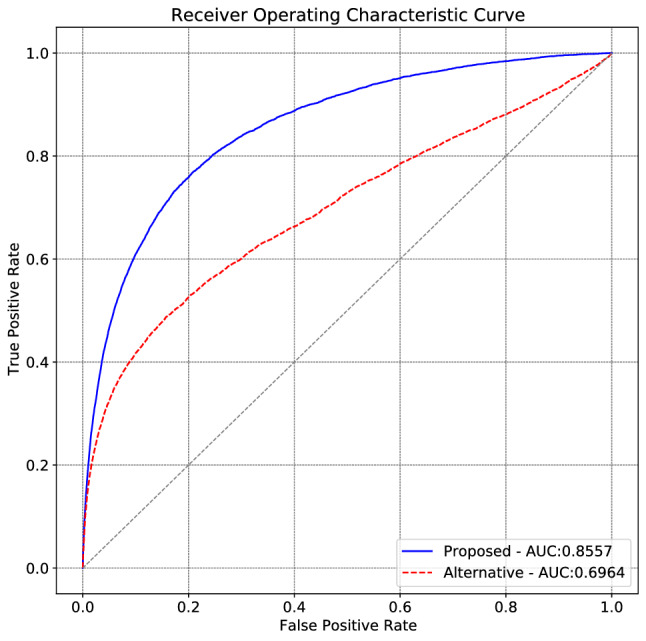
ROC curve and AUC score of the test set with the proposed and alternative models.

According to AUC scores and ROC curves shown in Fig. 3, our proposed model has shown superior performance compared to the alternative model, which can also be inferred from the shapes of the ROC curves. The ROC curve of our proposed model is above the alternative method and is closer to the top-left corner, meaning a shape that is closer to the perfect classifier than the alternative method.

**Figure 4:**
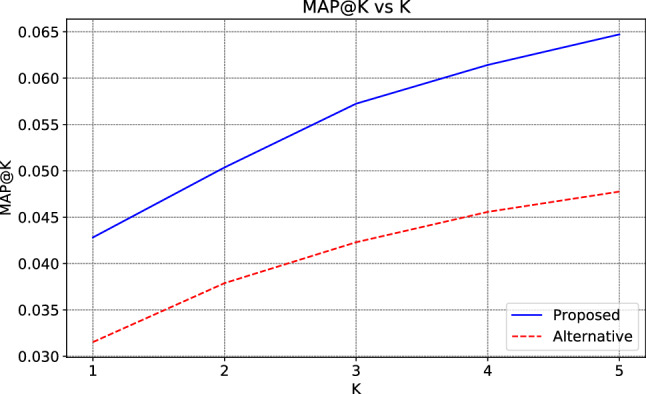
MAP@K Comparison of proposed and alternative models.

#### MAP@K

Higher *MAP*@*K* results show that the recommendation system can make more relevant recommendations. Figure 4 shows the *MAP*@*K* score for *K* values from 1 to 5 for both methods. Naturally, an increase in *K* (number of recommendations) will also increase *MAP*@*K*. For $K=5$, *MAP*@*K* values are 0.064718 and 0.047719 for the proposed and alternative methods. According to these values, the proposed model has shown 35% superior performance to the alternative method. Even if both models recommend the same merchants in their top 5 recommendations, the proposed method recommends the merchants in the higher ranks than the alternative method, measured by MAP@K precisely.

#### U-test statistics

Mann–Whitney U-Test^48^ is applied to evaluate the proposed and alternative methods in terms of their significance summarized in Table 8. The goal is to prove that prediction scores for positive links are significantly higher than negative ones. Since every combination of merchants and customers is considered in the test set, there are many negative links. We applied sampling to perform a reliable significance test. The test procedure is as follows: from the customers in the test, we selected ten people randomly, then positive and negative links of these people are taken. Positive links constitute the positive class directly, and 50 samples are randomly selected from negative links of these people and named as negative class. Then, the U-Test is applied between the positive and sampled negative classes of these ten people. This process (including taking ten people randomly) is repeated 1000 times.

**Table 8:** Significance comparisons of the proposed and alternative methods.

Model	Mean U-test stats.	Std U-test stats
Proposed	331.421	6.9475
Alternative	278.145	12.6452

**Figure 5:**
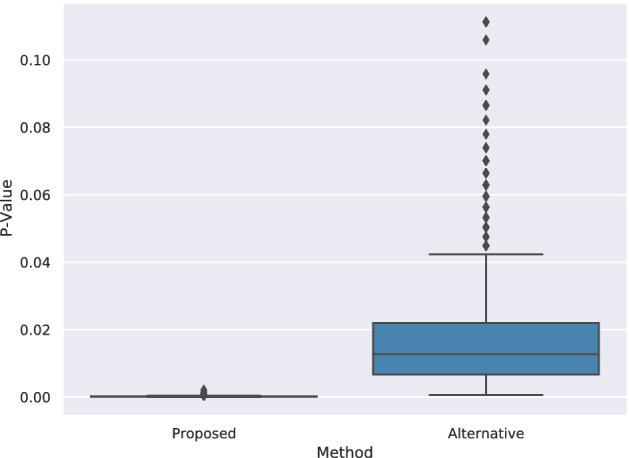
*p*-Value distribution distribution comparison.

**Figure 6:**
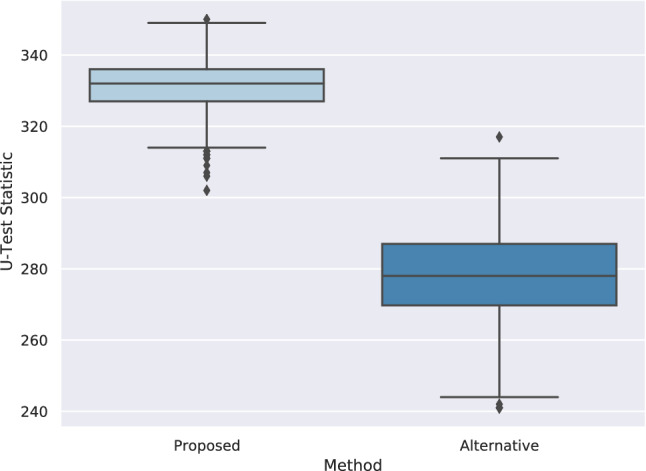
U-test statistic distribution comparison.

According to results presented in Fig. 5, *p*-values for both methods are (almost entirely) distributed in value ranges below 0.05, which means both methods produce statistically significant results. Since the *p*-value distribution shown in Fig. 5 of our proposed method is in smaller ranges, and its U-test statistics shown in Fig. 6 are higher than those of the alternative method, we can conclude that the proposed method has more significant results than the alternative one. The positive link prediction scores are significantly higher than the negative prediction scores, and obtaining these predictions by chance has a small probability.

#### Detection rates

Models are also compared based on their detection rates for five recommendations. According to results shown in Table 9, detection rate is the ratio of detected merchant purchases/visits in the top-5 recommendations to total merchants purchase/visits of two models. For example, the proposed method has caught 30.41% of the merchant purchases for this month compared to the alternative method at 22.03% in the top-5 recommendations, and the ratio of merchant purchases missed by the proposed model but caught by the alternative model is much less than the ratio for the other way around.

**Table 9:** Catch and Miss rates of top-5 recommendations of the proposed and alternative methods.

	Proposed model		
	Catch	Miss	Total
Alternative model			
Catch	0.1495	0.0708	0.2203
Miss	0.1546	0.6251	0.7797
Total	0.3041	0.6960	

#### Map comparison

**Figure 7:**
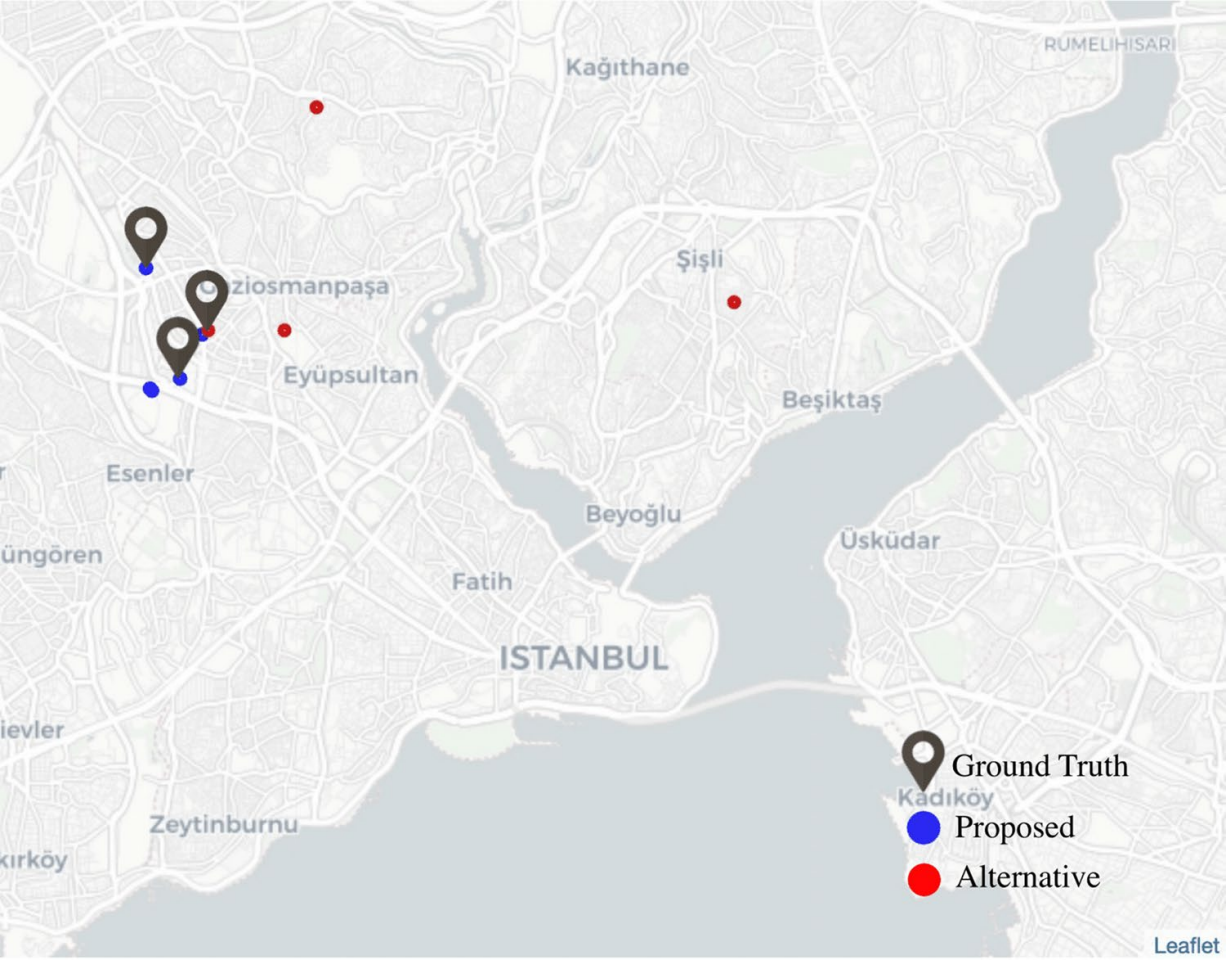
Map visualization of the recommendations by the proposed and alternative methods.

Recommendations of the two models for a sampled user are visualized on the map in Fig. 7. These visualizations include top-5 recommendations produced by the two models and this customer’s ground truth merchant visits. The ground truth merchants are shown as map markers, and the recommendations are shown as circles (blue for the proposed and red for the alternative method). According to the map, all of the three actual merchant visits are captured by the proposed method, and only one is caught by the alternative method. Also, the other two recommendations of the proposed method are much closer to the ground truths compared to those produced by the alternative method. As can be seen from Fig. 7, the proposed method makes more favorable predictions spatially as well as the alternative method. Therefore, we understand that the proposed method exploits the neighborhood information better.

## Discussion

In this study, we propose a link prediction framework to create a recommendation system for transaction datasets by using a binary classification approach that includes an embedding model and a classifier model. We examined several types of embedding and classifier models. Their performances are analyzed on a use case scenario, a merchant purchase prediction task in a credit card transaction dataset. After model and operator selection, we compare our proposed model with a collaborative filtering-based alternative method, the Alternating Least Squares^24^. According to comparative analysis, the proposed method shows superior performance to the alternative method in terms of MAP@K, AUC scores, and detection rates in the top-5 merchant recommendations. Overall, the proposed method demonstrates a convincing performance that can be used as a recommendation system. The items with the highest prediction scores can be utilized as top recommendations for a recommendation system.

Furthermore, our results confirm that using only previous interaction information can be a powerful signal that does not need or require other user or item-related features. Due to the robustness of the proposed method, one can use it with many different types of datasets, even when they are not in transactional form. For future work, one can further exploit the existence of the adjustable stages and high applicability of our proposed method, leading to improved models and/or implementations with other kinds of datasets. For instance, one can consider a weighted combination of the transactions’ time dimension and use the number of occurrences of user-item pairs to assign weights to network links while training the link embedding model. When examined for opportunities for further development, the proposed method currently operates only on the “seen” nodes, similar to many other link prediction techniques, and tries to discover the hidden links among the observed network, but it can be enhanced to incorporate unseen users and items by estimating their embeddings based on their external features^42^, which would help solve the “cold start” problem in recommendation systems.

Another way to improve the proposed method is by integrating a dimensionality reduction stage, which can help decrease training time and reduce overfitting. To further evaluate the performance of the method, it can be expanded to include use case scenarios such as product recommendations or song and movie recommendations based on previous purchase or listening/watching history data. Additionally, to improve scalability, reducing the number of pairwise combinations can be explored. This can be achieved by implementing a customer segmentation process using node features, such as location filters. The proposed method can make recommendations in certain neighborhoods, which can be used to infer location information and make new recommendations in these areas, thus reducing the number of pairwise combinations.

Our paper focuses on demonstrating the effectiveness of using transactional data for recommendation tasks by comparing it with the widely-used and established matrix factorization-based method, Alternating Least Squares (ALS). As part of our future work, we plan to conduct a survey paper that compares our method with other recommendation methods such as LightGCN^49^, a graph convolutional network architecture that has been proposed recently and addresses the sparsity and data heterogeneity problems commonly encountered in recommendation systems.

## Data Availability

he datasets generated during and/or analysed during the current study are not publicly available due to containing sensitive information that could compromise the privacy of participants in dataset but are available from the corresponding author on reasonable request.
